# Estimated plasma volume status (ePVS) is a predictor for acute myocardial infarction in-hospital mortality: analysis based on MIMIC-III database

**DOI:** 10.1186/s12872-021-02338-2

**Published:** 2021-11-08

**Authors:** Jun Chen, Jiayi Shen, Dongsheng Cai, Tiemin Wei, Renyi Qian, Chunlai Zeng, Lingchun Lyu

**Affiliations:** 1 Lishui Central Hospital, The Fifth Affiliated Hospital of Wenzhou Medical College, Lishui, 323000 Zhejiang China; 2 Zhejiang Chinese Medical University, Hangzhou, 310000 Zhejiang China; 3 Zhejiang University of Medical College, Hangzhou, 310000 Zhejiang China; 4 Department of Cardiology, Lishui Central Hospital and The Fifth Affiliated Hospital of Wenzhou Medical University, Lishui, 323000 China

**Keywords:** Acute myocardial infarction, Estimated plasma volume status (ePVS), Prognosis, MIMIC-III database

## Abstract

**Background:**

Estimated plasma volume status (ePVS) has been reported that associated with poor prognosis in heart failure patients. However, no researchinvestigated the association of ePVS and prognosis in patients with acute myocardial infarction (AMI). Therefore, we aimed to determine the association between ePVS and in-hospital mortality in AMI patients.

**Methods and results:**

We extracted AMI patients data from MIMIC-III database. A generalized additive model and logistic regression model were used to demonstrate the association between ePVS levels and in-hospital mortality in AMI patients. Kaplan–Meier survival analysis was used to pooled the in-hospital mortality between the various group. ROC curve analysis were used to assessed the discrimination of ePVS for predicting in-hospital mortality. 1534 eligible subjects (1004 males and 530 females) with an average age of 67.36 ± 0.36 years old were included in our study finally. 136 patients (73 males and 63 females) died in hospital, with the prevalence of in-hospital mortality was 8.9%. The result of the Kaplan–Meier analysis showed that the high-ePVS group (ePVS ≥ 5.28 mL/g) had significant lower survival possibility in-hospital admission compared with the low-ePVS group (ePVS < 5.28 mL/g). In the unadjusted model, high-level of ePVS was associated with higher OR (1.09; 95% CI 1.06–1.12; *P* < 0.001) compared with low-level of ePVS. After adjusted the vital signs data, laboratory data, and treatment, high-level of ePVS were also associated with increased OR of in-hospital mortality, 1.06 (95% CI 1.03–1.09; *P* < 0.001), 1.05 (95% CI 1.01–1.08; *P* = 0.009), 1.04 (95% CI 1.01–1.07; *P* = 0.023), respectively. The ROC curve indicated that ePVS has acceptable discrimination for predicting in-hospital mortality. The AUC value was found to be 0.667 (95% CI 0.653–0.681).

**Conclusion:**

Higher ePVS values, calculated simply from Duarte’s formula (based on hemoglobin/hematocrit) was associated with poor prognosis in AMI patients. EPVS is a predictor for predicting in-hospital mortality of AMI, and could help refine risk stratification.

**Supplementary Information:**

The online version contains supplementary material available at 10.1186/s12872-021-02338-2.

## Introduction

Acute myocardial infarction (AMI) is the most severe coronary artery disease and the leading cause of mortality worldwide, which accounts for almost 1.8 million annual deaths in Europe [1], and the estimated annual incidences of new and recurrent MI events in America are 0.55 million and 0.2 million events, respectively [2]. Although annual in-hospital mortality of AMI in Japan was decreased from 5.8% in 2006 to 5.2% in 2016 after adjusted age, this improvement was very slight [3]. How to reduce the in-hospital mortality of AMI further remains a problem that needs to be solved urgently. During the hospitalization of patients with AMI, fluid volume management is easy to overlook but an extremely important treatment strategy. However, whether the association between plasma volume (PV) and in-hospital mortality in AMI patients remains unclear, and there is no relevant research report until now.

Direct quantification of PV has been testified to have clinical utility to reveal volume overload severities in chronic heart failure (HF) patients, however, this methodology was not easy for clinicians to obtain [4]. Most hemodialysis devices use hematocrit to monitor volume during the hemodialysis process, but this method was relatively rough for assessing PV status [5]. Recently, Duarte et al. proposed a simple formula based on hematocrit and hemoglobin to estimate the plasma volume status [6]. A subsequent study confirmed that this formula has an acceptable fitness to the actual plasma volume state, which is measured by 125-I human serum albumin (concordance index 0.6, *P* < 0.01) [7]. In addition, Kobayashi et al. indicated that ePVS derived from Duarte formula was significantly associated with echocardiographic parameters of chronic HF patients [8].

Considering that PV status has essential value for the subsequent fluid volume management of AMI patients during the hospitalization, we aim to determine whether PV status derived from Duarte formula at hospital admission was associated with in-hospital mortality in AMI patients.

## Methods

### Data source

The primary data of our study was derived from MIMIC-III database. MIMIC-III database is an extensive and single-center database, constructed by Institutional Review Boards (IRB) of the Massachusetts Institute of Technology (MIT, Cambridge, MA, America) and Beth Israel Deaconess Medical Center. It contained more than 50,000 hospital patients admitted to intensive care units between 2001 and 2012 [9]. One of our authors (C.J, certification ID: 8979131) gained permission to documented the database after online training at the National Institutes of Health (NIH).

### Population selection

We included patients (aged > 18 years old) diagnosed with AMI at hospital admission by the International Classification of Diseases (ICD)-9 diagnosis codes between 410.00 and 410.52 in the MIMIC-III database. The diagnostic criteria of AMI were typical symptoms (defined as chest pain or dyspnea for < 6 h) and either ischemic changes on electrocardiography or elevated cardiac troponin on admission [10]. The exclusion criteria were: (I) Multiple admissions; (II) Missing survival outcome data; (III) During pregnancy and the postpartum period; (IV) Duration of hospital stay < 24 h; (V) Incomplete or unobtainable documented or other vital medical data records; (VI) Missing hemoglobin, hematocrit, weight, and height data.

### Clinical and laboratory data

Patients’ baseline characteristics (age, height, weight, diabetes history, hypertension history, liver disorders history, renal failure history) were collected. BMI was calculated as weight (kg) divided by height^2^ (m^2^). The first document of vital signs data and laboratory tests data of patients with AMI admitted to the hospital were extracted. Vital signs data included body temperature (T), systolic blood pressure (SBP), diastolic blood pressure (DBP), mean blood pressure (MBP), heart rate (HR), respiratory rate (RR), pulse oximetry derived oxygen saturation (spo2). Laboratory tests data included creatinine, anion gap, PH, blood urea nitrogen (BUN), chloride, glucose, hemoglobin, hematocrit, white blood cell count, platelet count, serum potassium, serum sodium, and activated partial thromboplastin time (APTT). Therapy included the use of vasoactive drugs (norepinephrine), percutaneous coronary intervention (PCI), coronary bypass graft surgery (CABG), and continuous renal replacement therapy (CRRT) during the hospitalization were also recorded. The simplified acute physiology score II (SAPSII) [11] and sequential organ failure assessment (SOFA) score [12] were also calculated for each patient. The endpoint of our study was in-hospital mortality which was defined as survival status at hospital discharge.

### Estimated plasma volume status equations

Two formulas were introduced to calculate the ePVS. The Duarte formula included hematocrit and hemoglobin as follows: ePVS (mL/g) = 100 × (1-hematocrit)/hemoglobin (g/dL) [6]. The Hakim formula was derived from hematocrit and dry body weight using the following equations. Actual plasma volume: (1-hematocrit) × (a + b × body weight in kg). Ideal plasma volume = c × body weight in kg. ePVS = [(actual plasma volume-ideal plasma volume)/ideal plasma volume] × 100 (males: a = 1530, b = 41.0, c = 39; females; a = 864, b = 47.9, c = 40) [13].

### Statistical analysis

Severe data (more than 20% data) missing was abandoned, and acceptable data missing was conducted with the multiple imputation [14]. The generalized additive model (GAM) was used to demonstrate the relationship between ePVS and all-cause hospital mortality, then select the cut-off value of ePVS according to the GAM for grouping. Continuous variables that exhibited a normal distribution were documented as the mean ± standard deviation (SD). Otherwise, they were documented as medians with upper and lower quartiles. Categorical variables were documented as frequencies with percentages. Groups comparison were pooled using the t-test or Wilcoxon rank-sum test for continuous variables, and the chi-square test or Fisher’s exact test for categorical variables. Kaplan–Meier survival analysis was used to pooled the difference of in-hospital mortality between the various groups and analyzed by Log-rank test. Variables based on epidemiological and laboratory test indicators may exist potential confounders [15]. Thus, three logistic regression models were introduced to adjusted those potential confounders. In the model I, covariates were mainly adjusted for vital signs data (age, gender, MBP, spo2) and comorbidities (hypertension, renal failure). In the model II, covariates were mainly adjusted for laboratory data (creatinine, PH, Glucose) based on the model I. In the model III, covariates were mainly adjusted for treatments (PCI, CRRT, CABG) based on the model II. Subgroup analysis of the association between ePVS and in-hospital mortality was performed using stratified logistic regression models. The discrimination of ePVS for in-hospital mortality was assessed by receiver operating characteristic (ROC) curve analysis. The area under the curve (AUC) of the ROC curve more than 0.7 was regarded as good discrimination, 0.65–0.70 represented acceptable discrimination. These results were expressed as odds ratio (OR) with 95% confidence intervals (CIs). All tests were 2-tailed tests, and *P* ≤ 0.05 was considered statistically significant. Statistical analyses were performed using R version 3.6.3 (R Foundation for Statistical Computing, Vienna, Austria).

## Results

### The characteristics of ePVS

**Fig. 1:**
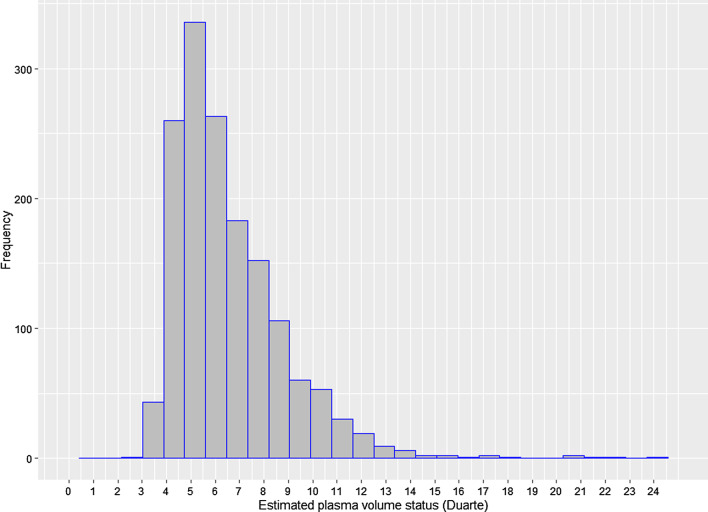
Distribution of ePVS in the entire study. ePVS: estimatedplasma volume status

**Fig. 2:**
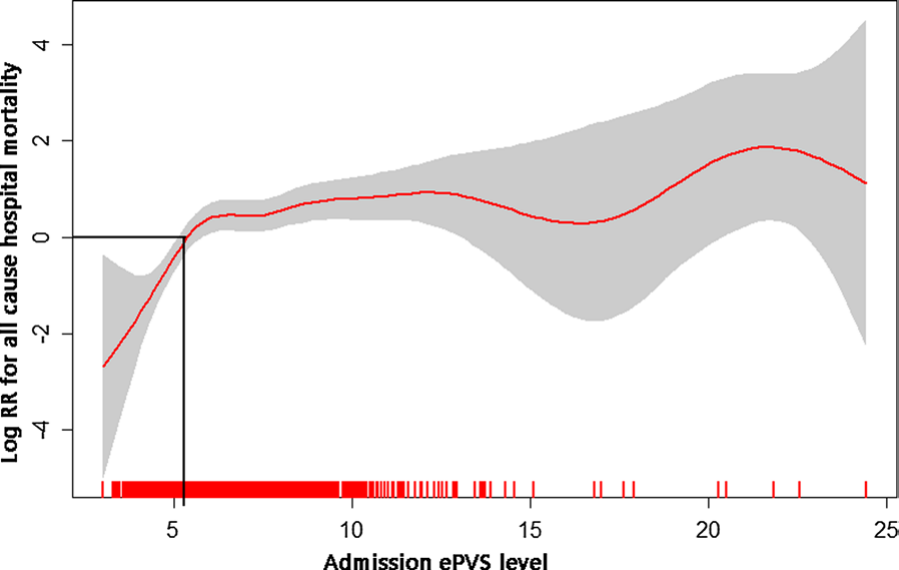
Cubic spline plot of relation of ePVS to risk of inpatient mortality. The model is fitted using restricted cubic splines with four knots in the generalized additive model. The ordinate represents log (RR) of in-hospital mortality. The abscissa represents the level of ePVS. The solid line represents the relationship between log (RR) of in-hospital mortality and admission ePVS level, and shaded area represents the 95% CI. When the log (RR) is 0, the corresponding ePVS level is used as the cut-off value. ePVS: estimated plasma volume status

**Fig. 3:**
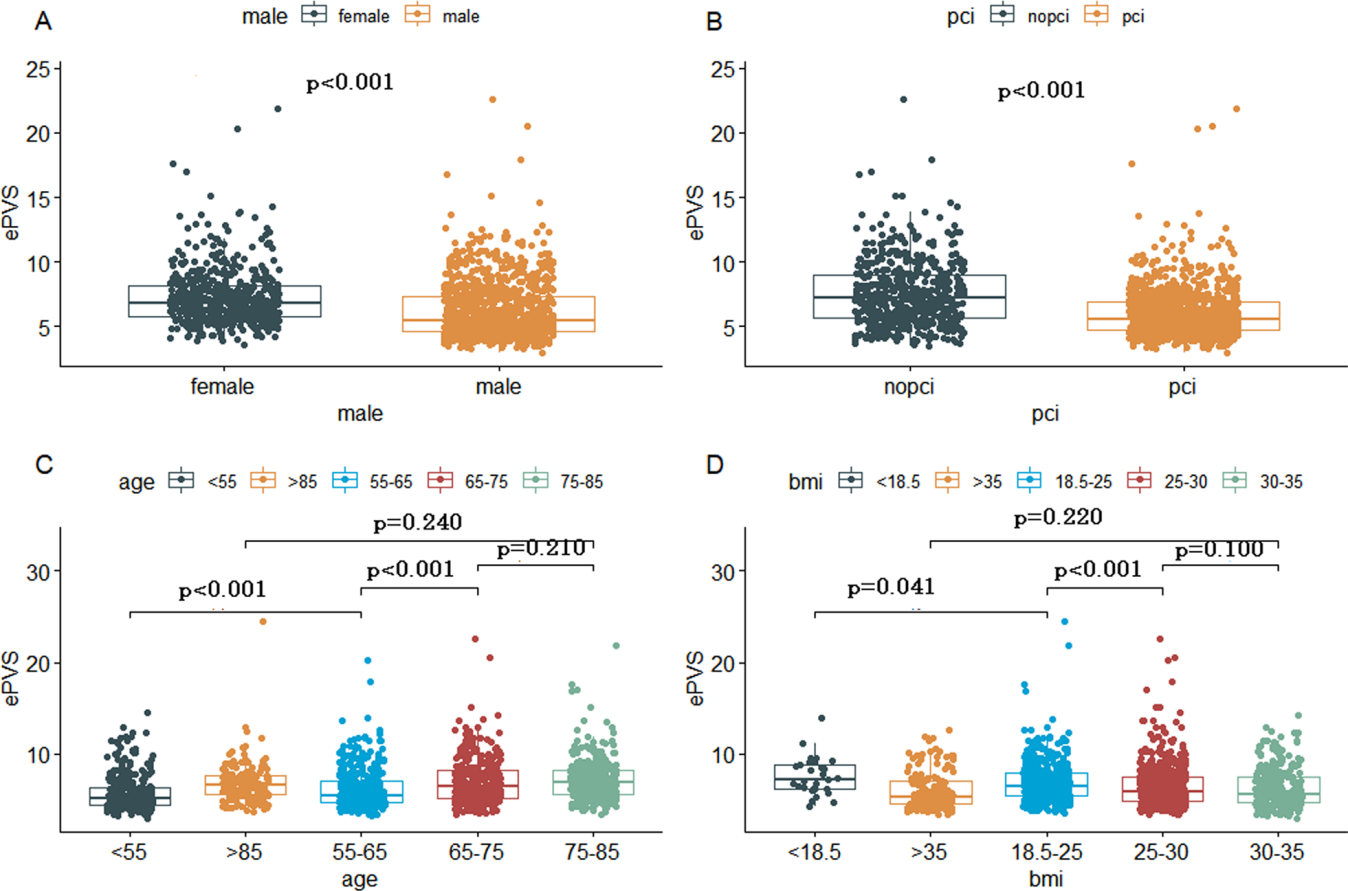
Comparison between different groups for ePVS. **A** the comparison of distribution differences of ePVS between man and women, **B** the comparison of distribution differences of ePVS between PCI group and NO-PCI group, **C** the comparison of distribution differences of ePVS among multiple ages, **D** the comparison of distribution differences of ePVS among multiple BMI

The distribution of ePVS was displayed in Fig. 1. The value of ePVS appeared skewed distribution and mainly concentrated in the range of 4–7 mL/g. The relationship between admission ePVS level and in-hospital mortality was nonlinear; higher ePVS was associated with increased in-hospital mortality; however, the mortality was not significantly increased when ePVS ≥ 5.28 ml/g, as shown in Fig. 2. In the study patients, ePVS was higher in female than the male with a significant difference (Fig. 3A). Compared with patients who undergo PCI, the patients who have not performed PCI showed a higher ePVS (Fig. 3B). The relationship between age and admission ePVS level showed that ePVS level increased significantly with age when age under 75 years old (Fig. 3C). Higher BMI tend to be associated with a lower level of ePVS (Fig. 3D).

### The characteristics of study patients

Totally, 1534 eligible patients (1004 males and 530 females) with an average age of 67.36 ± 0.36 years old were included in our study finally, more details about the data extraction process and missing data as shown in Additional file 1: Tables S1 and S2. 136 patients (73 males and 63 females) died in hospital, with the prevalence of in-hospital mortality was 8.9%. Patients were divided into two groups according to the GAM for ePVS and in-hospital mortality (the cut-off value of ePVS was 5.28 mL/g). The baseline characteristics of these patients were summarized in Table 1. The mean age for lower ePVS (ePVS < 5.28 mL/g) and higher ePVS (ePVS ≥ 5.28 mL/g) was 60.75 ± 0.59 and 70.28 ± 0.42 years old (*P* < 0.001). Patients with higher ePVS presented higher BUN level, higher creatinine level, higher SAPSII scores, and higher SOFA scores. The use of norepinephrine (17.99% vs. 5.48%) and CRRT (5.96% vs. 0.60%) were also more common in the higher-ePVS group than the lower-ePVS group. While, the higher ePVS group showed a lower SBP, DBP, BMI, glucose, and WBC count. In terms of surgical intervention, higher ePVS patients tend to undergo CABG (22.29% vs. 5.48%), however, lower ePVS patients tend to undergo PCI (82.0% vs. 33.53%). Also, patients with higher ePVS have a longer ICU and hospital stay than patients with the lower ePVS group.

**Table 1:** The characteristic of included subjects between different ePVS levels

Characteristic	Q1 (n = 511)	Q2 (n = 1023)	*P* value
Age (years old)	60.75 ± 0.59	70.28 ± 0.42	< 0.001
Man	443 (86.69%)	561 (54.83%)	< 0.001
BMI	29.62 ± 0.26	27.16 ± 0.17	< 0.001
Anion gap	13.29 ± 0.10	13.17 ± 0.10	0.390
SBP	116.38 ± 0.31	110.68 ± 0.45	< 0.001
DBP	66.72 ± 0.40	58.55 ± 0.29	< 0.001
MBP (mmHg)	82.30 ± 0.41	76.04 ± 0.29	< 0.001
Heart rate (beats/minute)	76.93 ± 0.59	82.35 ± 0.48	< 0.001
Respiratory rate (beats/minute)	18.28 ± 0.13	18.38 ± 0.11	0.6572
Temperature (°C)	36.78 ± 0.02	36.83 ± 0.02	0.215
SPO2 (%)	96.98 ± 0.10	97.54 ± 0.05	< 0.001
*Comorbidities, n (%)*			
Diabetes	116 (22.70%)	270 (26.39%)	0.246
Hypertension	177 (34.64%)	489 (47.80%)	0.002
Liver disease	12 (2.35%)	29 (2.83%)	0.708
renal_failure	18 (3.52%)	126 (12.32%)	< 0.001
*Laboratory parameters*			
BUN (mg/dL)	16.03 ± 0.31	21.02 ± 0.65	< 0.001
Bicarbonate	22.88 ± 0.15	21.54 ± 0.13	< 0.001
Creatinine (umol/L)	0.91 ± 0.02	1.23 ± 0.04	< 0.001
Chloride (mmol/L)	102.41 ± 0.17	102.69 ± 0.15	0.272
Glucose (mg/dL)	132.54 ± 2.00	119.33 ± 1.27	< 0.001
Hematocrit (%)	38.74 ± 0.12	28.77 ± 0.15	< 0.001
Hemoglobin (g/dL)	13.54 ± 0.04	9.84 ± 0.05	< 0.001
Platelet (10^9^/L)	223.66 ± 3.09	206.27 ± 2.93	< 0.001
PH	7.34 ± 0.004	7.32 ± 0.003	< 0.001
Potassium (mmol/L)	3.75 ± 0.02	3.74 ± 0.02	0.595
APTT (seconds)	37.40 ± 1.06	36.75 ± 0.62	0.474
Sodium (mmol/L)	137.06 ± 0.13	135.79 ± 0.13	< 0.001
WBC (10^9^/L)	11.66 ± 0.19	10.56 ± 0.14	< 0.001
*Scoring systems*			
SOFA	2.13 ± 0.10	4.01 ± 0.10	< 0.001
SIRS	2.36 ± 0.05	2.89 ± 0.03	< 0.001
LODS	2.33 ± 0.03	3.96 ± 0.09	< 0.001
SAPSII	26.00 ± 0.49	32.95 ± 0.56	< 0.001
*Treatment*			
norepinephrine	28 (5.48%)	184 (17.99%)	< 0.001
CRRT, n (%)	3 (0.60%)	61 (5.96%)	< 0.001
PCI	419 (82.0%)	343 (33.53%)	< 0.001
CABG	28 (5.48%)	228 (22.29%)	< 0.001
ICU LOS, days	2.94 ± 0.19	4.86 ± 0.23	< 0.001
HOS LOS (days)	5.23 ± 0.22	8.92 ± 0.29	< 0.001
HOS mortality, n (%)	16 (3.13%)	120 (11.73%)	< 0.001
30-day mortality, n (%)	24 (4.70%)	146 (14.27%)	< 0.001

*BMI* body mass index, *SBP* systolic blood pressure, *DBP* diastolic blood pressure, *MBP* mean blood pressure, *SPO2* Percutaneous oxygen saturation, *BUN* blood urea nitrogen, *APTT* activated partial thromboplastin time, *WBC* white blood cell, *CRRT* continuous renal replacement therapy, *SOFA* sequential organ failure assessment, *SAPSII* simplified acute physiology score II, *PCI* percutaneous coronary intervention, *CABG* coronary artery bypass grafting, *ICU* intensive care unit, *HOS* hospital, *LOS* long-term of stay

### EPVS levels and all-cause in-hospital mortality of AMI

**Fig. 4:**
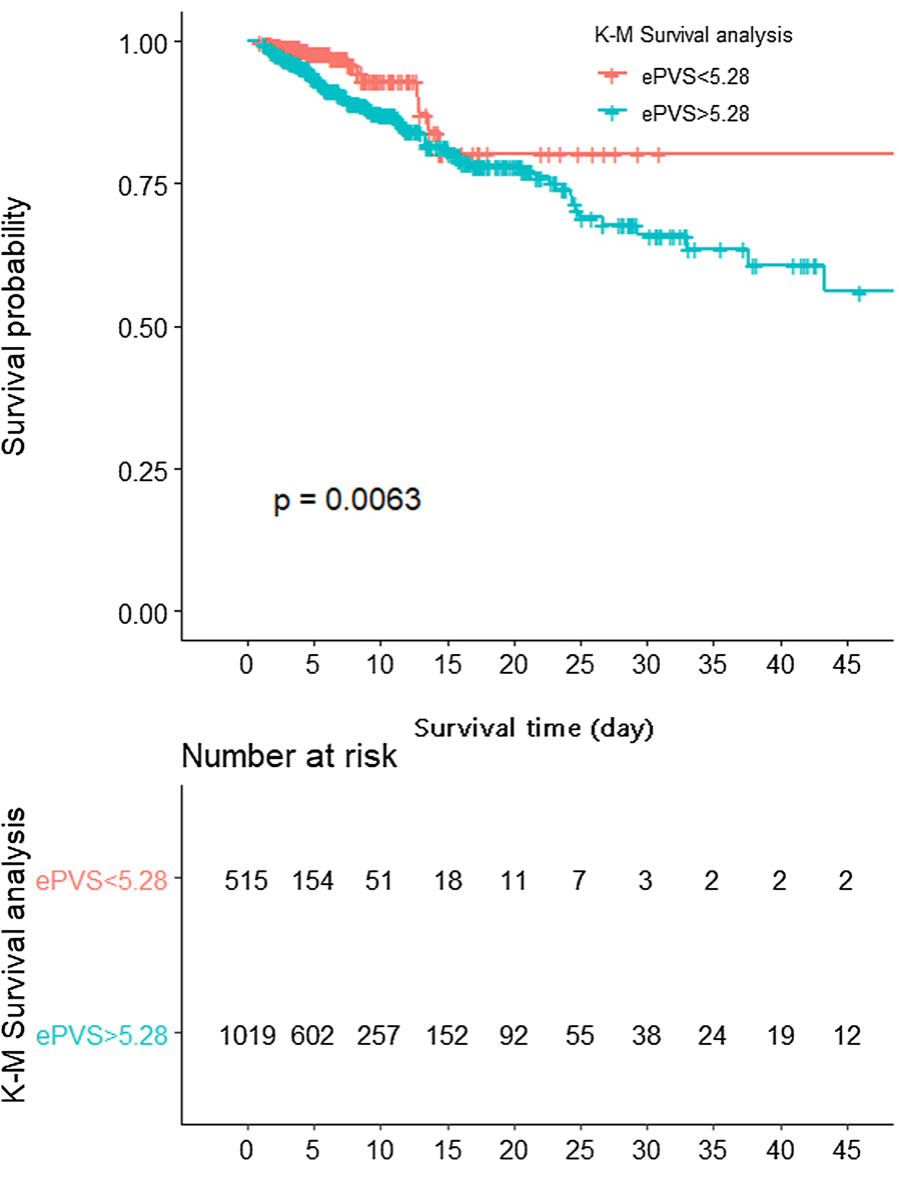
Kaplan–Meier survival curve for ePVS stratified by optimal cut-off. The result showed that the high-ePVS group (ePVS ≥ 5.28 mL/g) had lower survival possibility during the hospitalization than the low-ePVS group (ePVS < 5.28 mL/g), which reached statistical differences (log-rank test: *P* = 0.0063). ePVS: estimatedplasma volume status

In the unadjusted logistic regression model, high-level of ePVS (ePVS ≥ 5.28 mL/g) were associated with higher in-hospital mortality (OR 1.15; 95% Cl 1.09–1.22; *P* < 0.001) compared with low-level of ePVS (ePVS < 5.28 mL/g). We used three logistic regression models to determine the association between ePVS and in-hospital mortality in AMI patients after adjusted other confounding factors (Table 2). In model I, high-level of ePVS (ePVS ≥ 5.28 mL/g) was associated with increased risk of in-hospital mortality after adjusting vital signs data and comorbidities (OR 1.06; CI 1.03–1.10; *P* < 0.001). In model II, covariates were adjusted for laboratory data (creatinine, PH, Glucose) based on model I, high-level of ePVS also showed a significantly higher in-hospital mortality risk (OR 1.05; CI 1.01–1.08; *P* = 0.009). In model III, covariates were adjusted for treatments (PCI, CRRT, CABG) based on model II. The in-hospital mortality risk was also significantly higher in the high-ePVS group (OR 1.04; CI 1.01–1.07; *P* = 0.023). The survival curve for patients with different ePVS (ePVS ≥ 5.28 mL/g and ePVS < 5.28 mL/g) groups was shown in a Kaplan–Meier analysis plot in Fig. 4. The result showed that the high-ePVS group had lower survival possibility during the hospitalization than the low-ePVS group, which reached statistical differences (log-rank test: *P* = 0.0063).

**Table 2:** EPVS levels and all-cause in-hospital mortality of AMI

Variable	Unadjusted model		Model I		Model II		Model III	
	HR (95% CIs)	*P* value	HR (95% CIs)	*P* value	HR (95% CIs)	*P* value	HR (95% CIs)	*P* value
*In-hospital mortality*								
*ePVS*								
Q1	1.0 (ref)		1.0 (ref)		1.0 (ref)		1.0 (ref)	
Q2	1.09 (1.06–1.12)	< 0.001	1.06 (1.03–1.10)	< 0.001	1.05 (1.01–1.08)	0.009	1.04 (1.01–1.17)	0.023
*30 Day mortality*								
*ePVS*								
Q1	1.0 (ref)		1.0 (ref)		1.0 (ref)		1.0 (ref)	
Q2	1.10 (1.06–1.12)	< 0.001	1.06 (1.02–1.10)	0.002	1.04 (1.00–1.07)	0.047	1.03 (0.99–1.07)	0.105

Model I adjusted for: age, gender, mean blood pressure, SPO2, hypertension, renal failure

Model II adjusted for: Model I add creatinine, PH, Glucose

Model III adjusted for: Model II add CRRT, PCI, CABG

### Subgroup analyses

Among these strata, we observed that patients with higher ePVS had significantly higher in-hospital mortality in the PCI group (OR 1.08; CI 1.04–1.11; *P* < 0.001), the male group (OR 1.09; CI 1.06–1.12; *P* < 0.001), the absence of CRRT group (OR 1.08; CI 1.05–1.11; *P* < 0.001), and the absence of norepinephrine use group (OR 1.04; CI 1.02–1.07; *P* < 0.001). In the no-CABG group, patients with higher ePVS values were also associated with higher in-hospital mortality (OR 1.11; CI 1.07–1.14; *P* < 0.001) compared with lower ePVS value group. More details as seen Table 3.

**Table 3:** Subgroup analysis of the relationship between ePVS and all-cause in-hospital mortality

Characteristic	N	Q 1 (Ref)	Q 2	
			OR (95% CI)	*P* value
*Age (years old)*				
< 65	703	Ref	1.07 (1.04–1.10)	< 0.001
≥ 65	831	Ref	1.08 (1.02–1.14)	0.010
*Gender*				
Male	1008	Ref	1.09 (1.06–1.12)	< 0.001
Female	526	Ref	1.05 (0.97–1.15)	0.209
*BMI, kg/m*^*2*^				
< 27	723	Ref	1.09 (1.03–1.14)	0.002
≥ 27	811	Ref	1.09 (1.05–1.13)	< 0.001
*MBP, mmHg*				
< 75	619	Ref	1.07 (1.00–1.15)	0.049
≥ 75	915	Ref	1.08 (1.04–1.11)	< 0.001
*PCI*				
Yes	987	Ref	1.08 (1.04–1.11)	< 0.001
NO	547	Ref	1.07 (0.99–1.16)	0.070
*CABG*				
Yes	256	Ref	1.06 (0.97–1.15)	0.196
NO	1278	Ref	1.11 (1.07–1.14)	< 0.001
*CRRT*				
Yes	64	Ref	1.03 (0.58–1.81)	0.925
NO	1470	Ref	1.08 (1.05–1.11)	< 0.001
*Norepinephrine*				
Yes	212	Ref	1.10 (0.91–1.33)	0.335
NO	1322	Ref	1.04 (1.02–1.07)	< 0.001

*BMI* body mass index, *MBP* mean blood pressure, *CRRT* continuous renal replacement therapy, *PCI* percutaneous coronary intervention, *CABG* coronary artery bypass grafting

### The discrimination of ePVS for in-hospital mortality

**Fig. 5:**
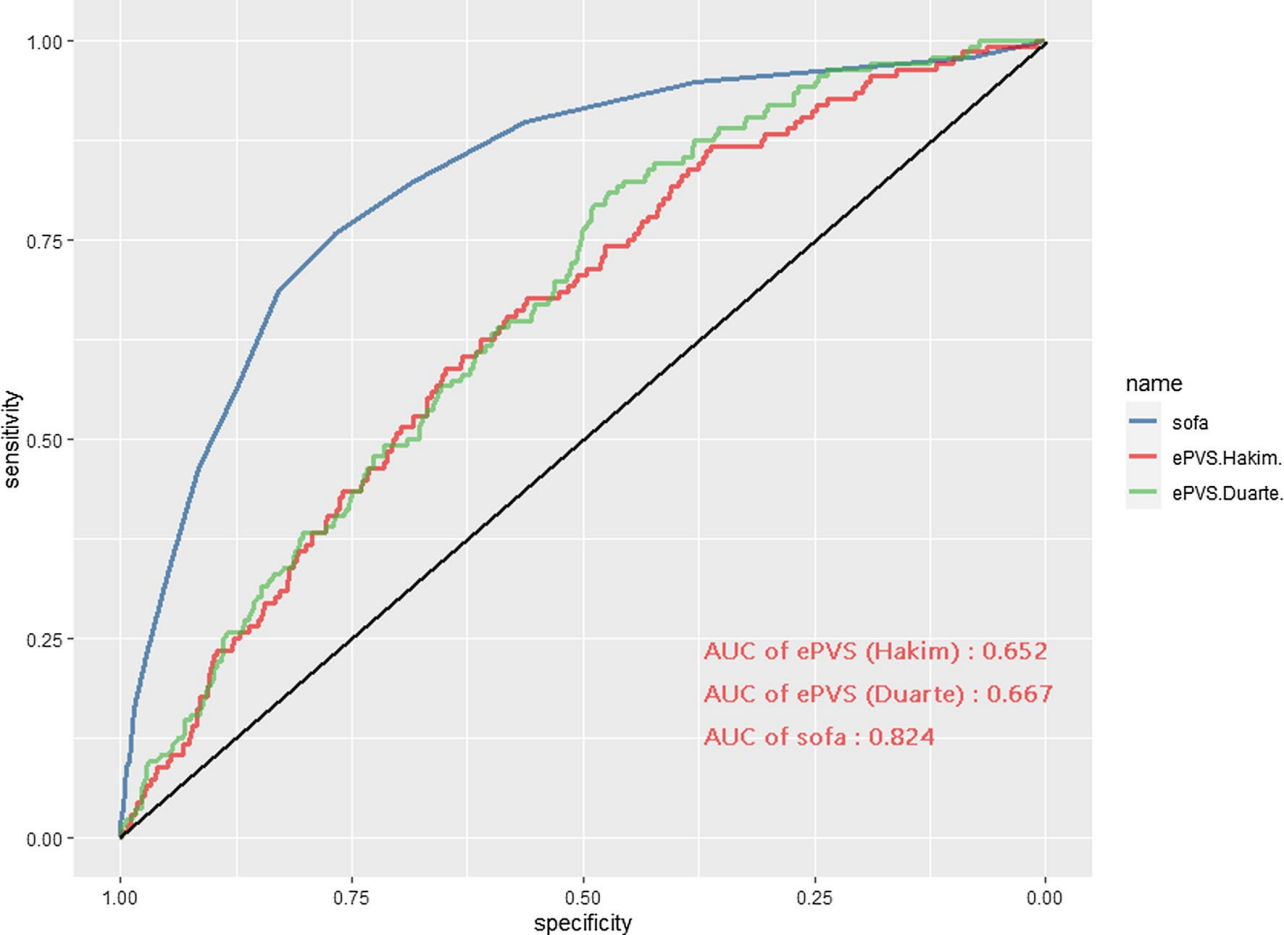
Receiver operating characteristic (ROC) curve of the nomogram. The area under the curve (AUC) of ROC was 0.667 (95% CI 0.653–0.681) for ePVS derived from Duarte formula. The area under the curve (AUC) was 0.652 (95% CI 0.641–0.663) for ePVS derived from Hakim formula. The area under the curve (AUC) was 0.824 (95% CI 0.789–0.859) for SOFA

The area under the curve (AUC) was 0.667 (95% CI 0.653–0.681) for Duarte-derived ePVS and 0.652 (95% CI 0.641–0.663) for Hakim-derived ePVS, it indicated that ePVS have an acceptable discrimination for predicting in-hospital mortality of AMI patients (Fig. 4). SOFA score was considered the best forecast scoring criteria for the in-hospital mortality of ICU patients [12]. The area under the curve (AUC) was 0.824 (95% CI 0.789–0.859) for the SOFA score (Fig. 5).

## Discussion

Our study demonstrated a “M-shaped” relationship between admission ePVS and in-hospital mortality among patients with AMI. The total in-hospital mortality was 8.9% in our study, which was similar to Yamamoto et al. study (5.2%) [3]. The present study investigated the relationship between Duarte-derived ePVS and prognostic of patients with AMI. Our main findings were: (1) higher-level of ePVS was independently associated with a higher risk of in-hospital death of patients with AMI; (2) Patients with higher-level ePVS have a significantly lower survival possibility during hospitalization than the patients with lower-level ePVS; (3) Duarte-derived ePVS and Hakim-derived ePVS have the similar accuracy for predicting in-hospital death of AMI patients; (4) Duarte-derived ePVS is also a promising biomarker for predicting in-hospital death of AMI patients who undergo PCI.

Previous researches have confirmed that ePVS derived from hemoglobin and hematocrit was independently associated with cardiovascular outcomes, rehospitalization, and death in acute or chronic HF [16–18]. Fudim et al. [19] reported that PVS was associated with in-hospital outcomes in decompensated HF. Duarte-ePV, trended towards an association with early decompensated HF clinical outcomes. Lin et al. [20] higher ePVS remained significantly associated with increased rate of primary outcome in chronic systolic HF (adjusted HR 1.567, 95% CI 1.267–1.936; *P* < 0.001). Two studies confirmed the clinical value of ePVS for predicting the prognosis of patients with fever [21], and the prognosis of patients with acute dyspnoea [22]. To our knowledge, there was only one study discuss the ePVS in AMI patients up to now. Maznyczka et al. found that higher ePVS (derived from Hakim formula) was associated with a worse prognosis of AMI patients who undergoing CABG, and the ePVS could help refine risk stratification for AMI patients who received CABG (AUC of ROC analysis:0.66, 95% CI 0.58–0.74, *P* < 0.001) [23]. However, our study showed that ePVS is a good biomarker for AMI patients’ prognosis after PCI rather than CABG. The insufficient sample size of CABG group patients in our study may be the major factor for the insignificant results. We found that Duarte-derived ePVS ≥ 5.28 mL/g is a risk factor for in-hospital death. After adjusted other influence factors, ePVS levels ≥ 5.28 mL/g were also linked to a 2–8% increased risk of 30-Day mortality and 5–1% increased risk of in-hospital mortality. Our findings outline certain underlying features of plasma volume that have not been previously addressed. Estimated plasma volume status was closely related to age. The elderly tend to present higher ePVS. Moreover, our study found that ePVS calculated by Duarte formula or calculated by Hakim formula have the similar accuracy for predicting in-hospital mortality of AMI patients. Compared with ePVS calculated by Hakim formula, Duarte-derived ePVS is easier to obtain just complete from the routine blood test. Therefore, Duarte-derived ePVS could become a quick and useful prognostic tool in patients with AMI.

The interpretation of high PV load state associated with the poor prognosis of AMI is currently unclear. Several potential interpretation should be taken into account. Coronary atherosclerotic heart disease patients with higher levels of ePVS may be accompanied by excessive activation of the renin–angiotensin–aldosterone system. Excessive neurohormones activation will cause water and sodium retention, leading to hemodynamic congestion and extravascular edema [16, 24, 25]. Furthermore, the excessive activation of the renin–angiotensin–aldosterone system would result in cardiac remodeling, which diminished cardiac function [26]. The oxygen supply–demand imbalance was the fundamental pathophysiology of AMI [27]. Reducing myocardial oxygen consumption (MVO2) during AMI restored the myocardial oxygen supply–demand balance in the presence of reduced supply and resulted in the reduction of infarct range [28]. Left ventricular (LV) mechanical work and heart rate were significant determinants of MVO2 [29]. In our study, the higher-level of ePVS group presented a higher heart rate (82.35 ± 0.48 vs. 76.93 ± 0.59, *P* < 0.001) compared with the lower-level of ePVS group. Thus, higher-level of ePVS means increased MVO2. Once AMI occurs, high PV load leads to increased myocardial oxygen consumption, progressive ventricular dilatation, and increased wall stress, which can exacerbate myocardium ischemia. On the other hand, higher levels of ePVS may be a manifestation of disease severity. Recently, the PARADISE registry study, which enrolled 1369 patients admitted for acute dyspnoea in the emergency department, found that ePVS values greater than 5.12 mL/g presented an adjusted odds ratio of 1.47 (95% CI 1.04–2.09, *P* = 0.029) for in-hospital mortality [22]. Marawan et al. [30] study showed that high ePVS was associated with an increased risk of all-cause death, with a hazard ratio of 1.29 (95% CI 1.24–1.25, *P* < 0.001), this results was similar to our findings. In our study, the higher-level of ePVS patients gained higher SAPSII (32.95 ± 0.56 vs. 26.00 ± 0.49, *P* < 0.001) and SOFA score (4.01 ± 0.10 vs. 2.13 ± 0.10, *P* < 0.001) compared with lower-level of ePVS ones. At the same time, higher levels of ePVS group presented a higher rate of renal failure (12.32% vs. 3.52%, *P* < 0.001), and CRRT (5.96% vs. 0.60%, *P* < 0.001) compared with lower-levels of ePVS group. It suggested that congestion during AMI may be the critical factor for further aggravation of myocardial ischemia, and excessive congestion was associated with renal function. However, Kobayashi et al. [17] study did not find any statistical interaction between ePVS and estimated glomerular filtration rate (eGFR). Their study suggested that there was no association between Duarte-derived ePVS and renal function. The specific relationship between ePVS and renal function needs further researches to confirm. Our study showed that AMI patients with the negative fluid balance on admission have better clinical outcomes. Restrictive fluid administration and a consequent early negative fluid balance were associated with lower in-hospital mortality. These results have important implications for clinical practice. We need to closely monitor the PV load during hospitalization of patients with AMI, and guide their follow-up fluid treatment according to the PV load. PV estimated from hemoglobin/hematocrit using Duarte’s formula, may represent a quick and useful marker in patients with AMI in clinical routine.

### Limitations

There were several limitations in our study. Firstly, this study was based on a observational study, and the sample size was not large enough. Therefore, large prospective cohorts study are needed to confirm our findings in the future. Secondly, our study only focused on the ePVS derived from the first laboratory test on admission with inevitable bias. Future studies could research the in-hospital change trend of the ePVS of patients with AMI, and further explore the role of fluid volume management in AMI. Thirdly, several potential confounding variables (albumin, BNP) due to severe data missing were unable to assess. Given that, external validation was required to test its utility. Fourth, our study included patients with AMI, however, we did not made further subdivision for AMI with STEMI or NSTEMI, STEMI have different pathological processes compared with NSTEMI. Finally, specific target ePVS goals was 5.28 mL/g in our study, but it is unclear whether the same targets should be applied to different subgroup patients.

## Conclusion

In conclusion, a higher ePVS value, calculated simply from Duarte’s formula (based on hemoglobin/hematocrit) was associated with poor prognosis in AMI patients. Duarte-derived ePVS would be a promising biomarker for predicting the prognosis of patients with AMI. Restrictive fluid administration is an essential treatment strategy for the management of AMI. Further study of ePVS in AMI patients may yield opportunities to reduce the in-hospital mortality risk of AMI patients and improve patient outcomes. Further investigations are warranted to evaluate the potential clinical utility of ePVS guided management in patients with AMI.

## Supplementary Information

**Additional file 1. Tables S1:** The extraction process of present study data; **Tables S2**: The details of missing data.

## Data Availability

The clinical data used to support the findings of this study were supplied by Monitoring in Intensive Care Database III version 1.4 (MIMIC-III v.1.4). Although the database is publicly and freely available, researchers must complete the National Institutes of Health’s web-based course known as Protecting Human Research Participants to apply for permission to access the database. Data are available to researchers on request for purposes of reproducing the results or replicating the procedure by directly contacting the corresponding author.

## Abbreviations

GAM: Generalized additive model
ICD: International classification of diseases
ICU: Intensive care unit
ePVS: Estimated plasma volume status
AMI: Acute myocardial infarction
HF: Heart failure
SBP: Systolic blood pressure
DBP: Diastolic blood pressure
MBP: Mean blood pressure
HR: Heart rate
MIMIC: Medical information mart for intensive care
SPO2: Pulse oximetry derived oxygen saturation
WBC: White blood cell count
HR: Hazard ratio
SOFA: Sequential organ failure assessment
CABG: Coronary bypass graft surgery
BUN: Blood urea nitrogen
APTT: Activated partial thromboplastin time
PCI: Percutaneous coronary intervention
CRRT: Continuous renal replacement therapy
SAPSII: Simplified acute physiology score II
AUC: Area under the curve
ROC: Receiver operating characteristic
PV: Plasma volume
