# Supplementary material for: Estimated plasma volume status (ePVS) is a predictor for acute myocardial infarction in-hospital mortality: analysis based on MIMIC-III database
Source: BMC Cardiovasc Disord. 2021 Nov 8;21:530. doi: 10.1186/s12872-021-02338-2 (PMC8573972; doi:10.1186/s12872-021-02338-2)
Supplement: Supplementary file 1 — Additional file 1. Tables S1: The extraction process of present study data; Tables S2: The details of missing data. [file 12872_2021_2338_MOESM1_ESM.docx]

**Table S1 Number of observations after each selection procedure**

| Step | Criteria | Number excluded | Number of remaining observations |
| --- | --- | --- | --- |
| 1 | ICU admissions in MIMIC-III database | - | 58,976 |
| 2 | Include patients on admission diagnosed with AMI (ICD-9 code between 410.00 and 410.52) | - | 1,626 |
| 3 | Exclude patients with multiple admissions except for the first admission | 32 | 1,594 |
| 4 | Include if patients aged ≥18 years | 0 | 1,594 |
| 5 | Exclude if length of hospital stay <24 hours | 40 | 1,554 |
| 6 | Exclude if patients with missing hematocrit data information | 20 | 1,534 |

**Table S2 Missing number (%) for characteristics**

| Characteristics | Missing number (%) |
| --- | --- |
| Demographics |  |
| Age | 0 |
| Gender | 0 |
| Ethnicity | 0 |
| Marital status | 0 |
| height | 196 (12.8%) |
| weight | 56 (3.7%) |
| BMI | 213 (13.9%) |
| Vital signs |  |
| HR | 23 (1.5%) |
| SBP | 23 (1.5%) |
| DBP | 23 (1.5%) |
| MBP | 23 (1.5%) |
| RR | 23 (1.5%) |
| T | 43 (2.8%) |
| SpO2 | 23 (1.5%) |
| Comorbidities |  |
| diabetes | 0 |
| metastatic_cancer | 0 |
| Laboratory parameters |  |
| Anion gap | 47 (3.1%) |
| albumin | 881 (57.4%) |
| bicarbonate | 33 (2.2%) |
| bilirubin | 714 (46.5%) |
| creatinine | 27 (1.8%) |
| chloride | 28 (1.8%) |
| glucose | 22 (1.4%) |
| hematocrit | 0 |
| hemoglobin | 0 |
| lactate | 882 (57.5%) |
| potassium | 19 (1.2%) |
| aptt | 118 (7.7%) |
| inr | 121 (7.9%) |
| pt | 126 (8.2%) |
| sodium | 22 (1.4%) |
| bun | 25 (1.6%) |
| wbc | 29 (1.8%) |
| platelet | 26 (1.6%) |
| Scoring system |  |
| SOFA | 0 |
| SIRS | 0 |
| LODS | 0 |
| QSOFA | 0 |
| SAPSII | 0 |
| Renal replacement treatment | 0 |
| ICU LOS | 0 |
| In-hospital LOS | 0 |
| In-hospital mortality | 0 |
